# miRNAs Copy Number Variations Repertoire as Hallmark Indicator of Cancer Species Predisposition

**DOI:** 10.3390/genes13061046

**Published:** 2022-06-10

**Authors:** Chiara Vischioni, Fabio Bove, Matteo De Chiara, Federica Mandreoli, Riccardo Martoglia, Valentino Pisi, Gianni Liti, Cristian Taccioli

**Affiliations:** 1 Department of Animal Medicine, Production and Health, University of Padova, 35020 Legnaro, Italy; chiara.vischioni@phd.unipd.it; 2 IRCAN, CNRS, INSERM, Université Côte d’Azur, 06107 Nice, France; matteo.dechiara@unice.fr (M.D.C.); gianni.liti@unice.fr (G.L.); 3 Department of Physics, Informatics and Mathematics, University of Modena and Reggio Emilia, 41125 Modena, Italy; fabio.bove.dr@gmail.com (F.B.); federica.mandreoli@unimo.it (F.M.); riccardo.martoglia@unimo.it (R.M.); valentino.pisi@gmail.com (V.P.)

**Keywords:** DNA copy number variation, miRNAs, comparative study

## Abstract

Aging is one of the hallmarks of multiple human diseases, including cancer. We hypothesized that variations in the number of copies (CNVs) of specific genes may protect some long-living organisms theoretically more susceptible to tumorigenesis from the onset of cancer. Based on the statistical comparison of gene copy numbers within the genomes of both cancer-prone and -resistant species, we identified novel gene targets linked to tumor predisposition, such as CD52, SAT1 and SUMO. Moreover, considering their genome-wide copy number landscape, we discovered that microRNAs (miRNAs) are among the most significant gene families enriched for cancer progression and predisposition. Through bioinformatics analyses, we identified several alterations in miRNAs copy number patterns, involving miR-221, miR-222, miR-21, miR-372, miR-30b, miR-30d and miR-31, among others. Therefore, our analyses provide the first evidence that an altered miRNAs copy number signature can statistically discriminate species more susceptible to cancer from those that are tumor resistant, paving the way for further investigations.

## 1. Introduction

Aging is considered one of the risk factors of cancer insurgence due to the mutational burden derived from cell division and DNA replication [1]. Therefore, it is probable that, in order to maintain a high longevity rate, those organisms that live longer should theoretically possess a higher risk of cancer occurrence. Nevertheless, considering different species, according to Peto’s Paradox theory [2], the body size of an organism and/or its lifespan expectation are not directly correlated with the species percentage of cancer incidence. After more than 40 years of research, the solution to this puzzling paradox is still an open challenge to be solved. For example, despite its small size, the naked mole rat is, to date, the longest-living member of the rodent family, being able to live more than 30 years. Several studies highlighted that, besides the delayed aging, this species also shows the capacity to resist spontaneous and experimentally induced tumorigenesis [3,4,5,6]. Conversely, in some other rodents, the cancer-related mortality can reach 90%, coupled with a species maximum life expectancy of 4–5 years [7]. The long-living *Myotis lucifugus* bat species has been recently identified as a prospective organism for comparative cancer research [8]. Given their extended life-span rates [9], it has been suggested that bats develop a very low number of cancer events, as confirmed by different pathological studies performed in different areas of the world [10,11]. The elephant has been pinpointed as another cancer-resistant species [12], with a cancer incidence rate considerably lower compared to the human one, for example (approximately 22%) [13]. In order to maintain a high longevity, some species might have developed intrinsic molecular mechanisms that protect them from cancer onset or development [14]. Interestingly, various authors recently reported that the genome of the African elephant encodes multiple copies of the TP53 gene, also known as the “guardian of the genome stability”. This amplification could be at the basis of the elephant’s anti-cancer and longevity mechanisms by leading to increased levels of apoptosis in response to DNA damage [12,15]. Indeed, according to Caulin and Maley (2011) [16], the genome of large long-living organisms can reveal an altered number of tumor suppressors and oncogenes (in multiple or reduced copies), which might represent a possible mechanism underlying their capacity of exceeding the threshold of cancer onset, despite their phenotypic predisposition due to body size and longevity [16]. Copy number variations (CNVs) are duplications or deletions of genomic regions which can be associated with phenotypic alterations, including tumorigenic diseases [17]. In particular, a variation in the gene copy numbers can influence the activity of tumor suppressors and oncogenes, leading to the development of cancer [18]. Within this framework, long-living animals have to rely on compensatory mechanisms to suppress and prevent cancer progression, which can be straightened by different molecular and genomic mechanisms such as altered gene copy numbers that increase the number of tumor suppressors paralogues or reduce copies of oncogenes [19,20]. As previously mentioned, mammals have evolved lifespan and cancer incidence rates that vary among species [21], but the mechanisms underlying these differences are still unclear. In order to test the hypothesis that genomic CNVs are related to the cancer incidence rate of a species, we compared the genome-wide copy number landscapes of nine different mammals (five cancer-resistant and four cancer-prone species) and identified the target genes that can significantly discriminate between these two groups. Contrary to what is usually done, we did not use an a priori list of cancer-related genes but included all human genes in our analysis dataset. In this way, we were able to identify miRNAs, usually removed from evolutionary comparative analyses, as the most enriched elements able to discriminate those organisms that are predisposed to cancer from those that are not.

## 2. Materials and Methods

### 2.1. Data Collection

According to the hypothesis that positively selected CNVs tend to recur during cancer progression [22,23], but also during the evolution [24], we have recently developed the VarNuCopy database, a unique database that collects the CNVs landscape for multiple organisms, with the aim to compare patterns of copy number changes across the genome of different species [25]. We used a homemade script written in Perl 5.14 and Python 3 in order to download the CNV data from Ensembl comparative genomics resources (http://www.ensembl.org accessed on 1 March 2019) [26], an ideal system to perform and support vertebrate comparative genomic analyses, given the consistency of gene annotation across the genomes of different vertebrate species. We leveraged Ensembl’s “gene gain/loss tree” feature, which displays the number of copies of extant homologous genes for each species in a taxonomic tree view [27]. These data are estimated through CAFE (Computational Analysis of gene Family Evolution), a computational tool commonly used to study gene family evolution, which takes into account a priori the species phylogenetic tree [27,28]. The Perl API script provided by the Ensembl website was used to access the genomic databases and used to download all the available CNVs data. We encoded a new homemade Python script to arrange the CNVs data counts in a readable tab-delimited format and used this matrix to perform the subsequent analysis.

### 2.2. Statistical Comparison

Using a comparative approach, we analyzed the variation landscape of the gene copies among the genomes of nine organisms sub-set in two categories: “cancer resistant” (*Heterocephalus glaber* (Hg), *Nannospalax galili* (Ng), *Dasypus novemcinctus* (Dn), *Loxodonta africana* (La) and *Myotis lucifugus* (Ml)) and “cancer prone” (*Mus musculus* (Mm), *Rattus norvegicus* (Rn), *Canis familiaris* (Cf) and *Homo sapiens* (Hs)) species (Supplementary Table S1). We classified as “cancer resistant” those species that, based on the literature review, are known to possess a low cancer incidence rate. Conversely, “cancer prone” organisms are those species for which the percentage of tumors found in a certain number of necropsies is known to be high.

**Figure 1:**
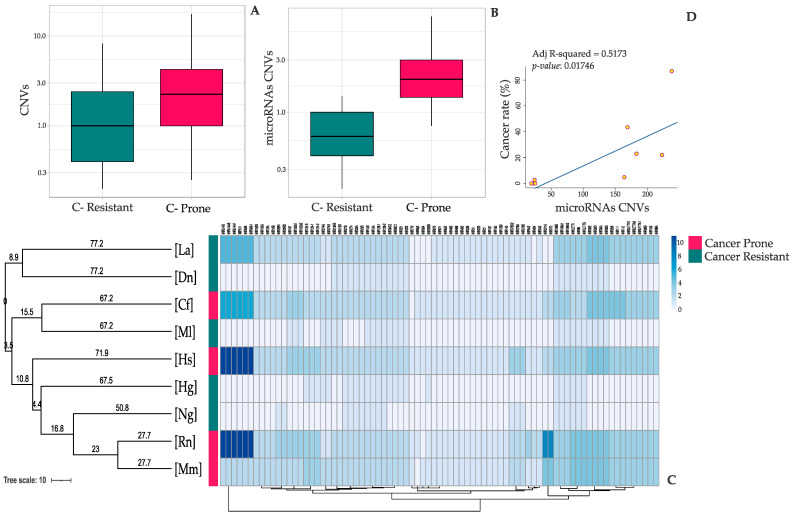
CNV landscape comparisons: (**A**) Boxplot of the distribution of significant gene CNVs in cancer-prone vs. cancer-resistant species. (**B**) Boxplot of the distribution of significant microRNA CNVs in cancer-prone vs. cancer-resistant species. Cancer-resistant species are highlighted in green, cancer-prone species in red. In the boxplots, the *Y*-axis scale has been changed to log one. The boxplots were built considering the average number of copies of each gene in the two different target groups. (**C**) Heatmap representing the microRNA CNV repertoires within the nine analyzed species—(Hg): *Heterocephalus glaber*; (Ng): *Nannospalax galili*; (Dn): *Dasypus novemcinctus*; (La): *Loxodonta Africana*; (Ml): *Myotis lucifugus*; (Mm): *Mus musculus*; (Rn): *Rattus norvegicus*; (Cf): *Canis familiaris*; (Hs): *Homo sapiens*. Hg, Ng, Dn, La and Ml have been previously described as cancer-resistant species. Mm, Rn, Cf and Hs are known to be cancer-prone species. Phylogeny was inferred from VertLife [35], created and visualized through the Interactive Tree of Life web-tool [36]. (**D**) PGLS correlating the cancer incidence rate with the total number of significant microRNAs copies across the nine species included in the analysis. The blue line represents a positive correlation between the two variables (adjusted R^2^ = 0.5173; *p*-value = 0.01746).

Cancer incidence rate data were collected from different recently published literature [4,5,6,8,10,11,12,15,21,29,30,31,32,33]. We performed a statistical comparison between the CNVs of the two different species groups, cancer-prone and -resistant organisms, with the aim to identify new possible gene targets able to discriminate between the two categories. Thus, a statistical unpaired two-group Wilcoxon test was performed using R.3.1.1(R Foundation for Statistical Computing, Vienna, Austria), to compare their entire CNV spectra. To determine whether microRNAs CNVs independently contribute to the variation in cancer incidence percentages among our species, we applied a linear regression model through the PGLS R package [34], in order to check for potential bias due to species phylogeny or population structure (Figure 1D). The phylogenetic tree included in the analysis was derived from VertLife [35] and created and visualized through the Interactive Tree of Life web-tool (Figure 1C) [36]. Data processing and statistical tests were performed with R.3.1.1. Figures were made using the ggplot2 R package, in association with different R Shiny apps such as BoxPlotR, PlotsOfData, ClustVis, and miRTargetLink 2.0 [37,38,39,40].

### 2.3. Pathways Analysis

To determine if the CNVs are enriched in specific gene families, we used Gene SeT AnaLysis Toolkit, a tool for the interpretation of lists of interesting genes that is commonly used to extract biological insights from targets of interest [41]. The set of significant genes were tested for pathway associations using the hyper-geometric test for over-representation analysis (ORA) [42] (Supplementary Table S4). We selected different pathway enrichment categories (KEGG: https://www.genome.jp/; Wikipathway: https://www.wikipathways.org; Reactome: https://reactome.org/; PANTHER: http://www.pantherdb.org/ accessed on 1 June 2019), considering as over-represented those molecular networks with an FDR significance level lower than 0.05, after a correction with the Benjamini–Hochberg method. In this context, the ORA analysis was the preferred option among the others (e.g., gene set enrichment or network topology-based analysis) in order to obtain biological information underlying the significantly enriched genes, resulting in a reduction in the complexity of the data interpretation [42].

## 3. Results

A two-group comparison was performed using a Wilcoxon rank sum test, in order to identify an existing distinction in the distribution of the number of gene copies between cancer-prone and cancer-resistant species. A list of the most significant hits (*p*-value < 0.05), including known tumor suppressors and oncogenes, is reported in Table 1 (see Supplementary Tables S2 and S3 for the extended version). Our analysis, which exclusively considered the variation in number of gene copies among different species, was able to identify those genes involved in biological processes related to cancer development and maintenance.

### 3.1. Best Candidate Cancer-Related Genes

**Figure 2:**
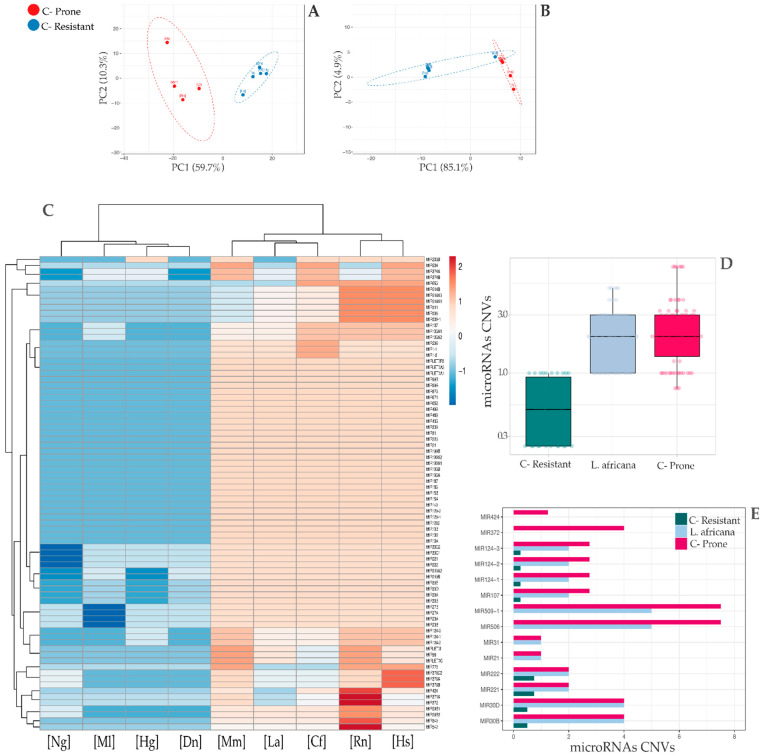
(**A**) PCA based on the CNVs of all the significant genes. (**B**) PCA based on the CNVs of the significant microRNAs subset. Both plots show a dichotomy between cancer-resistant (blue) and cancer-prone species (red). (**C**) Heatmap of the significant microRNAs, clustered with Euclidean distance and complete linkage. (**D**,**E**) Bar and box plots of the significant microRNAs CNVs in cancer-prone species, cancer-resistant species and *Loxodonta africana*. The microRNAs repertoire of *Loxodonta africana* seems to reflect the cancer-prone miRNAs copy number alteration landscape, rather than the one typical of the cancer-resistant organisms. In the box plots, the *Y*-axis scale was changed to log one. The boxplots are built considering the average number of copies of each gene in the two different target groups.

The distribution of the average number of each gene copies plotted in Figure 1A highlights a difference between the two species categories, which appears even greater if we only refer to the microRNAs CNVs landscape (Figure 1B). Among the most significant genes presenting an altered number of copies, we found CD52 (*p*-value = 0.007), SAT1 (*p*-value = 0.007), DMD (*p*-value = 0.014), EIF5 (*p*-value = 0.017), SUMO2, SUMO3, SUMO4 (*p*-value = 0.024), S100A16 (*p*-value = 0.030), MBD1, MBD2, MBD3 (*p*-value = 0.031), FGFBP1 (*p*-value = 0.032), FOXJ1 (*p*-value = 0.032), NUPR1 (*p*-value = 0.032), SELENOW (*p*-value = 0.032) and JUND (*p*-value = 0.034). Some of these, such as DMD, MDB1, NUPR1 and JUND, have been already well described as tumor suppressors or oncogenes [47,60,67,70], whereas the others do not officially belong to any of these two categories and they have been proposed as key regulators in biological processes such as cell proliferation, migration and cancer development and progression [42,43,48,52,53,59,61,62,68,69]. A Principal Component Analysis (PCA) of the CNV values of the nine species reported in Figure 2A,B showed a clear dichotomy between the cancer-prone and -resistant groups, supporting the hypothesis that an altered CNV landscape is able to discriminate between the two categories. To confirm these results, we performed another unsupervised clustering analysis using Euclidean distance (Figure 2C).

As depicted in the heatmap, each cluster has a distinct set of copy number values, and the main branches representing cancer-prone and -resistant organisms perfectly distinguish the two groups. No additional information (other than copy numbers) was given to the algorithm. In addition, we applied the Euclidean distances, using both the ‘complete’ and ‘ward’ methods (criteria that direct how the subclusters are merged) (Supplementary Figures S2–S4)). Remarkably, using this method, the *Loxodonta africana* microRNAs CNV landscape seems to have a different pattern as compared to the other cancer-resistant species (Figure 2C), confirming the elephant as an outlier species of the cancer-resistant group (see Section 4).

### 3.2. Cancer-Related MicroRNAs Pathways Are among the Most Significantly Enriched Biological Families

Our analysis shows an enrichment of onco-miRNAs amplifications in the cancer-prone species group. In particular, miR-424 (*p*-value = 0.009), miR-372 (*p*-value = 0.010), miR-107 (*p*-value = 0.022), miR-124 (*p*-value = 0.022), miR-506 (*p*-value = 0.029), miR-511 (*p*-value = 0.029), miR-378A (*p*-value = 0.030), miR-1 (*p*-value = 0.032), miR-206 (*p*-value = 0.032) and miR-340 (*p*-value = 0.032) are few examples of the most significant microRNA hits, which possess a suppressor and/or oncogenic role (Figure 1C). Given the high diversity among our species, we used the generalized least squares (PGLS) phylogenetic method [34] in order to assess whether copy number and cancer incidence rates evolved in a dependent manner along the tree, or if their relationship might be the consequence of common ancestry, resulting in similar patterns of miRNAs copy number alteration. Indeed, taking into account the genetic structure of the population, the PGLS comparative method confirmed the association between these traits independently of the shared evolutionary history of the species (Figure 1D and Supplementary File S1).

### 3.3. ORA Analysis Confirms a Significant Enrichment in the miRNAs Gene Family

We performed an Over-Representation Analysis (ORA) [41] on the complete list of significant genes, in order to identify enriched functional categories potentially related to cancer (Table 2 and Supplementary Figure S1). The most enriched pathways outputted by the ORA analysis were “MicroRNAs in cancer”, “miRNAs involved in DNA damage response”, “Metastatic brain tumor”, “miRNA targets in ECM and membrane receptors”, “let-7 inhibition of ES cell reprogramming” and “miRNAs involvement in the immune response in sepsis” [72,73]. These results indicate that the genes more prone to CNVs were those encoding miRNAs involved in cancer initiation, chronic inflammation and immune response. Remarkably, performing the ORA analysis applying the PANTHER algorithm [74], we also found a significant enrichment in the “Cadherin signaling network”, which is a well-known molecular pathway described as a key player in cancer [75].

## 4. Discussion

Being theoretically more susceptible to cancer, big and long-living species need additional cancer defense molecular mechanisms. On the other hand, short-living and small-size organisms might not need them because of their lower intrinsic predisposition to cancer due to their short lifespan rate. CNVs can therefore be considered one of the multiple protection ways against tumor insurgence that can explain Peto’s Paradox. In fact, we hypothesized that all cancer-resistant organisms implemented a series of molecular mechanisms to counteract their cancer predisposition, which depends on and derives from their own specific evolutionary history. We believe that CNVs that increase the onco-suppressive capacity of specific genes can be an excellent defense against tumor diseases in species at risk. Indeed, some authors have recently suggested that one of the most effective cancer-resistance strategies is represented by an augmentation in the number of copies of tumor suppressor genes [76]. In contrast, a reduced cancer-resistance rate could be caused by a selective loss of the same suppressor genes [77]. For instance, the CD52 gene (higher number of copies in the cancer prone group), a membrane glycoprotein expressed on the surface of mature lymphocytes, monocytes and dendritic cells, was one of the most significant hits of our analysis (*p*-value = 0.007). Recently, Wang and co-authors [43] identified CD52 as a key player in tumor immunity, affecting tumor behavior by regulating the associated tumor microenvironment. With the same significant *p*-value of 0.007, we also identified the SAT1 gene (higher number of copies in the cancer prone group) as one of the possible targets to be further investigated in the context of tumor onset. This gene can regulate and drive brain tumor aggressiveness, promoting molecular pathways that act in response to DNA damage and regulation of the cell cycle [44]. Another significant gene resulting from our analysis was represented by the SUMO protein family members (higher number of copies in the cancer resistant group). During cell cycle progression, many tumor suppressors and oncogenes are regulated via SUMOylation [78], a biological process that, if deregulated, can lead to genome instability and altered cell proliferation. In this context, it is evident that some tumors could be dependent on the functional SUMO pathway, but whether it is required for tumor growth remains to be established. For this reason, SUMO2, SUMO3 and SUMO4 can be potentially exploited in further anti-cancer mechanisms investigations (*p*-value = 0.024 in the present study), in order to shed light on the regulatory mechanisms underlying the activity of SUMO machinery in an oncogenic framework. Among the most significant hits, we also retrieved some genes that are already known to be tumor suppressors or oncogenes (DMD and JUND, respectively). Indeed, mutation or deregulated expression of Duchenne Muscular Dystrophy gene (DMD) is often linked to the development and progression of some major cancer types [48], such as sarcomas, carcinomas, melanomas, lymphomas and brain tumors [79,80], being a well-known tumor suppressor in different types of human cancers. On the other hand, JUND, a member of the AP-1 family that is related to MYC signaling pathway, regulates cell cycle and proliferation and its overexpression is linked to many types of cancers (PCA i.e.,) [71].

Notably, our results show that miRNAs are the most enriched gene family in discriminating between cancer-prone and cancer-resistant species. The specific role of these miRNAs is not yet fully understood, but we speculate that some of them might possess important regulatory functions aimed at defending some species (big size and long lifespan organisms) from cancer, while, at the same time, they are capable of exposing others to tumorigenesis (small-size and short-lifespan mammals). MicroRNAs (miRNAs) are small post-transcriptional molecular regulators that are able to modify gene expression levels, increasing the amount of mRNA degradation or inhibiting protein translation [81]. Since each single miRNA can regulate the expression of dozens of genes, many authors were able to correlate their activity with cell development, homeostasis and disease [82], including cancer [83,84]. Indeed, some tumorigenic events are caused by a malfunction in the heterogeneous regulatory activity of microRNAs inside the eukaryotic cells. Depending on the specific tissue and on the relationship with the immune system, they can behave both as tumor suppressors and as oncogenes [85]. Furthermore, epigenetic factors and species genetic predisposition can drive their double-sided behavior, although some of them are evolutionarily conserved within vertebrate taxonomic families [86]. Several miRNAs have already been described in the literature as oncogenes and tumor suppressors. For example, miR-424 is known to be a human tumor suppressor that can inhibit cell growth enhancing apoptosis or suppressing cell migration [45]. MiR-372, instead, can participate in WNT cancer molecular pathway [46], whereas the overexpression of miR-107, mediating p53 regulation of hypoxic signaling, can suppress tumor angiogenesis and growth in mice [50]. MiR-1 is another example of tumor suppressor microRNA that has been previously found to be significantly down-regulated in squamous carcinoma cells [64]. MiR-30b and miR-30d are considered suppressors in tumors that do not affect immune cells, whereas they have been found to be upregulated in melanoma [87]. In a similar way and for the first time, our analysis revealed several miRNAs candidates that might be involved in a mammalian species cancer predisposition (Figure 1C).

Interestingly, all the miRNAs that we have found show many more copies in the cancer-prone group as compared to the cancer-resistant species, and most of them are well-known oncogenes (miR-221, miR222, and miR-372, etc.). MiR-372, for instance, is not present in cancer-resistant species, whereas it shows multiple copies in those ones belonging to the cancer-prone group. This microRNA plays a pivotal role in the initiation of breast cancer and may activate the WNT and E2F1 pathways during the epithelial–mesenchymal transition process [46,47]. We also found an amplification of miR-221 and miR-222 in the cancer-prone category. Previous literature has extensively described these two RNAs as oncogenes, being deregulated in primary brain tumors and in acute lymphoid leukemia, among other malignancies [88,89]. According to our results, surprisingly, cancer-prone species showed the amplification of miR-15 tumor suppressor, which is known to be able to regulate cancer proliferation initiation by targeting the BCL2 gene [90,91]. Our hypothesis is that this apparent paradox may underlie a defensive role of this microRNA in those species that are, a priori, susceptible to tumor insurgence. On one hand, according to the so-called “gene dosage hypothesis”, gains or losses of specific gene copies can have a dramatic impact on the fitness of a species, leading to altered phenotypes due to the change in the expression levels of the affected genes [92]. On the other hand, oncogenes amplification or tumor suppressors deletions are not always detrimental, but can recapitulate tumorigenic events, being drivers or modulators of the disease [93]. As mentioned before, in fact, differences in ecology and evolutionary history are believed to give rise to significant differences between short- and long-living animals [94], and consequently in cancer-prone and -resistant species. In 2020, Tollis and co-authors [20] showed that mammalian lifespan can be correlated to both suppressor gene and oncogene CNVs, a phenomenon that they themselves called “paradoxical”. Interestingly, our analysis also leans in the same direction, suggesting that when high copy numbers of oncogenes shorten a lifespan, they must somehow be counterbalanced by higher number of copies of tumor-suppressor genes.

In this framework, the elephant’s miRNAs amplification signature resembles that of the organisms of the cancer-prone group (Figure 2D,E). In fact, it showed an alteration in the copy numbers of known oncogenes, such as miR-221 and miR-222, together with miR-30b/d and miR-31. In our opinion, *Loxodonta africana* should be placed in a new category of organisms, which share both oncogenic and cancer-resistant characteristics, being also clustered as an outlier species of the cancer-resistant group (Figure 2B). During their evolution, elephants may have selected certain molecular mechanisms, such as the amplification of TP53 and pseudogenes [12,15], with the aim to defend their cells from the tumorigenic action of a high percentage of onco-miRNAs copy number amplification and high longevity. Consequently, an additional amplification in the number of tumor suppressor microRNAs would have not been sustainable/useful in terms of fitness and/or survival. The hypothesis is that species with bigger sizes and longer lifespans have an expanded number of tumor-suppressor genes (TSGs), which is even higher than the one of their oncogenic counterparts. In this way, the direct elimination of oncogenes, which implies elevated costs in terms of growth and cellular functions maintenance, can be avoided, thus reducing the cancer incidence risk. In support of this, Vazquez and Lynch (2021) [76] reported that, within the Afrotheria order, the tumor-suppressor genes found in an altered number of copies were relatively lower compared to what might be expected. This finding can mirror the trade-off mechanism that natural selection has developed during evolution in order to compensate for the multi-copies effect that can lead to an increased risk of cancer, due to the unbalanced number of copies of the same genes. Indeed, long-living species might possess mechanisms that are capable of maintaining the equilibrium between proliferation and tumor control. Their regulatory networks can create positive feedbacks in which the amplification of tumor suppressor families functions as a buffer against the oncogene co-expansion, or vice-versa [20]. On the other hand, the cancer-prone organisms included in our analysis did not develop these gene defenses because they have a lower lifespan, which does not make them particularly exposed to a severe lack of fitness due to cancer progression (except in the case of *Homo sapiens* that has reached a high lifespan only recently, thanks to the advance of medicine treatments and health care).

## 5. Limitations and Future Perspectives

Gene duplication is a fundamental process that can lead to the emergence of new phenotypic traits. Analyzing patterns of gene copy number alterations across the genome of large and long-living organisms may reveal new insights about the mechanisms underlying cancer resistance in mammals [12,20,94]. Here, we have developed a simple way to test the hypothesis that CNVs confer protection or increase vulnerability to cancer among species. Using the absolute number of copies of each gene by species, we were able to identify, for the first time, an alteration in miRNA CNVs that are overrepresented and enriched in molecular pathways related to cancer. Further analyses will help to validate these findings by better defining the correlation between miRNAs and their targets. Nowadays, the current challenge is to develop and optimize new experimental design and strategies to be used in human [95] and veterinary biomedical research. Indeed, whenever a potential cancer-suppression mechanism is discovered in a species, there is the real possibility of identifying a new molecular target or therapeutic approach. Therefore, the investigation of genomic alterations, such as CNVs, can direct clinical research towards the discovery of new toolkits able to guide scientists towards the exploration of more focused research topics, such as, for example, specific microRNAs or their targets [96,97].

## Figures and Tables

**Table 1:** Genomic CNV landscape comparisons. Subset of 25 significant hits resulting from the unpaired 2-group Wilcoxon test (*p*-value < 0.05). The statistical comparison was made in order to identify those genes able to discriminate between the cancer-prone and -resistant species groups, relying exclusively on the genomic copy number values. Some of these genes are already known to be tumor suppressor and/or oncogenes, whereas the others can play pivotal roles in tumorigenesis events, and, for this reason, can be considered as targets to be further investigated and validated in the context of cancer development.

Gene	*p*-Value	Known_TS	Known_OG	References
CD52	0.007	NO	NO	[43]
SAT1	0.007	NO	NO	[44]
MIR424	0.009	YES	NO	[45]
MIR372	0.010	NO	YES	[46,47]
DMD	0.014	YES	NO	[48]
EIF5	0.017	NO	NO	[49]
MIR107	0.022	YES	YES	[50,51]
MIR124-1, MIR124-2, MIR124-3	0.022	YES	NO	[52]
SUMO2, SUMO3, SUMO4	0.024	NO	NO	[53,54]
MIR506	0.029	YES	YES	[55]
MIR509-1	0.029	NO	NO	[56]
MIR511	0.029	YES	NO	[57]
MIR514A1, MIR514A3, MIR514B	0.029	NO	NO	[58]
MIR378A	0.030	YES	NO	[59]
S100A16	0.030	NO	NO	[60]
MBD1, MBD2, MBD3	0.031	NO	YES (MDB1)	[61]
FGFBP1	0.032	NO	NO	[62]
FOXJ1	0.032	NO	NO	[63]
MIR1-1, MIR1-2	0.032	YES	NO	[64]
MIR206	0.032	YES	NO	[65]
MIR340	0.032	YES	NO	[66]
MIR542	0.032	NO	NO	[67]
NUPR1	0.032	YES	NO	[68]
SELENOW	0.032	NO	NO	[69,70]
JUND	0.034	NO	YES	[71]

**Table 2:** Pathway analysis. Gene Over-Representation Analysis (ORA) using KEGG, PANTHER and Wikipathway. The enrichment test used Benjamini–Hochberg’s FDR correction (FDR < 0.05). CNV data were previously analyzed by an unpaired 2-group Wilcoxon test (*p*-value < 0.05). Significant genes altered in their number of copies within the entire genomic landscape were used to perform the ORA analysis, which highlighted a significant enrichment in microRNAs and cancer-related pathways.

	Description	FDR (BH)	Genes
**KEGG**	MicroRNAs in cancer	0	MIR103A1; MIR103A2; MIR107; MIR124-1; MIR124-2; MIR124-3; MIR1-1; MIR1-2; MIR206; MIR100; MIR10A; MIR10B; MIR129-1; MIR129-2; MIR15A; MIR15B; MIR193B; MIR199A1; MIR199A2; MIR199B; MIR203B; MIR21; MIR223; MIR31; MIR99A; MIRLET7A1; MIRLET7A3; MIRLET7F2; MIR29B1; MIR29B2; MIRLET7G; MIRLET7I; MIR221; MIR222; MIR23A; MIR23B; MIR27A; MIR27B; MIR30C1; MIR30C2; MIR30A; MIR30B; MIR30D; MIR30E.
	Taste transduction	3.16 × 10^−10^	TAS2R10; TAS2R13; TAS2R14; TAS2R19; TAS2R20; TAS2R3; TAS2R30; TAS2R31; TAS2R42; TAS2R43; TAS2R45; TAS2R46; TAS2R50; TAS2R7; TAS2R8; TAS2R9
	Progesterone-mediated oocyte maturation	2.43 × 10^−4^	SPDYE1; SPDYE11; SPDYE16; SPDYE17; SPDYE2; SPDYE2B; SPDYE3; SPDYE4; SPDYE5; SPDYE6; INS
	Oocyte meiosis	2.73 × 10^−4^	PPP3R2; SPDYE1; SPDYE11; SPDYE16; SPDYE17; SPDYE2; SPDYE2B; SPDYE3; SPDYE4; SPDYE5; SPDYE6; INS
**PANTHER**	Cadherin signaling pathway	4.02 × 10^−2^	PCDHB14; PCDHB7; PCDHGB1; PCDHB16; PCDHB6; PCDHGB4; PCDHGA6; PCDHGB6; PCDHGB7
**Wikipathway**	miRNAs involved in DNA damage response	3.76 × 10^−9^	MIR371A; MIR372; MIR542; MIR100; MIR15B; MIRLET7A1; MIR374B; MIR221; MIR222; MIR23A; MIR23B; MIR27A; MIR27B
	Alzheimers Disease	5.31 × 10^−5^	MIR124-1; MIR124-2; MIR124-3; MIR10A; MIR129-1; MIR129-2; MIR199B; MIR21; MIR433; MIR671; MIR873; PPP3R2; MIR29B1; MIR30C2; MIR219A2
	Metastatic brain tumor	2.31 × 10^−3^	MIRLET7A1; MIRLET7A3; MIRLET7F2; MIR29B1; MIR29B2; MIRLET7G
	miRNA targets in ECM and membrane receptors	2.31 × 10^−3^	MIR107; MIR15B; MIR30C1; MIR30C2; MIR30B; MIR30D; MIR30E
	MicroRNAs in cardiomyocyte hypertrophy	2.77 × 10^−3^	MIR103A1; MIR103A2; MIR140; MIR15B; MIR185; MIR199A1; MIR199A2; MIR23A; MIR27B; MIR30E
	Cell Differentiation - Index	1.25 × 10^−2^	MIR1-1; MIR206; MIR199A1; MIR199A2; MIR221; MIR222
	let-7 inhibition of ES cell reprogramming	1.25 × 10^−2^	MIRLET7A1; MIRLET7F2; MIRLET7G; MIRLET7I
	miRNAs involvement in the immune response in sepsis	1.43 × 10^−2^	MIR187; MIR199A1; MIR199A2; MIR203B; MIR223; MIR29B1; MIRLET7I
	Cell Differentiation-Index expanded	2.38 × 10^−2^	MIR1-1; MIR206; MIR199A1; MIR199A2; MIR221; MIR222
	Role of Osx and miRNAs in tooth development	3.35 × 10^−2^	MIRLET7A1; MIRLET7F2; MIR29B1; MIRLET7G; MIRLET7I

## Data Availability

All data necessary for confirming the conclusions of the article are present within the article, figures, tables, and its Supplementary Materials.

## Supplementary Materials

The following are available online at https://www.mdpi.com/article/10.3390/genes13061046/s1. Table S1: Species description. List of the 9 species included in our analysis; Table S2: Cancer Prone vs. Cancer Resistant: a two-group statistical comparison. List of the significant hits resulting from the unpaired 2-group Wilcoxon test (*p*-value < 0.05) applied on the genomic CNVs landscape of the selected species; Table S3: Cancer Prone vs Cancer Resistant: a two-group statistical comparison. List of the significant microRNAs resulting from the unpaired 2-group wilcoxon test (*p*-value <0.05) applied on the total genomic CNVs landscape of the selected species; Table S4: Pathway analysis—extended version. Gene Over-Representation Analysis (ORA). The enrichment test used Benjamini-Hochberg’s FDR correction (FDR < 0.05). CNVs data were previously analyzed by an unpaired 2-group wilcoxon test (*p*-value < 0.05); Figure S1: MicroRNAs in cancer interaction graph. Figure S2: Heatmap of all the significant genes, clustered with Euclidean distance and ward linkage; Figure S3: Heatmap of all the significant genes, clustered with Euclidean distance and complete linkage; Figure S4: Heatmap of the significant MicroRNAs, clustered with Euclidean distance and ward linkage; File S1: PGLS modelling results: Cancer incidence rate ~ significant miRNAs CNVs.
