# Supplementary material for: miRNAs Copy Number Variations Repertoire as Hallmark Indicator of Cancer Species Predisposition
Source: Genes (Basel). 2022 Jun 10;13(6):1046. doi: 10.3390/genes13061046 (PMC9223155; doi:10.3390/genes13061046)
Supplement: Supplementary file 1 [file genes-13-01046-s001.zip › Vischioni_et_al_Supplmentary_2022.pdf]

Supplementary File S1:

Statistical results of the PGLS model correlating cancer incidence rate ~ total number of significant microRNAs copies across the 9 species included in the analysis, applied in order to check for potential bias due to species phylogeny or population structure.

model <- pgls(cancer_rate ~ CNV, lambda="ML", data = comp.data)

pgls(formula = cancer_rate ~ CNV, data = comp.data, lambda = "ML")

Coefficients:

- Residual standard error: 2.235 on 7 degrees of freedom
- Multiple R-squared: 0.5776,
- Adjusted R-squared: 0.5173
- p-value: **0.01746**

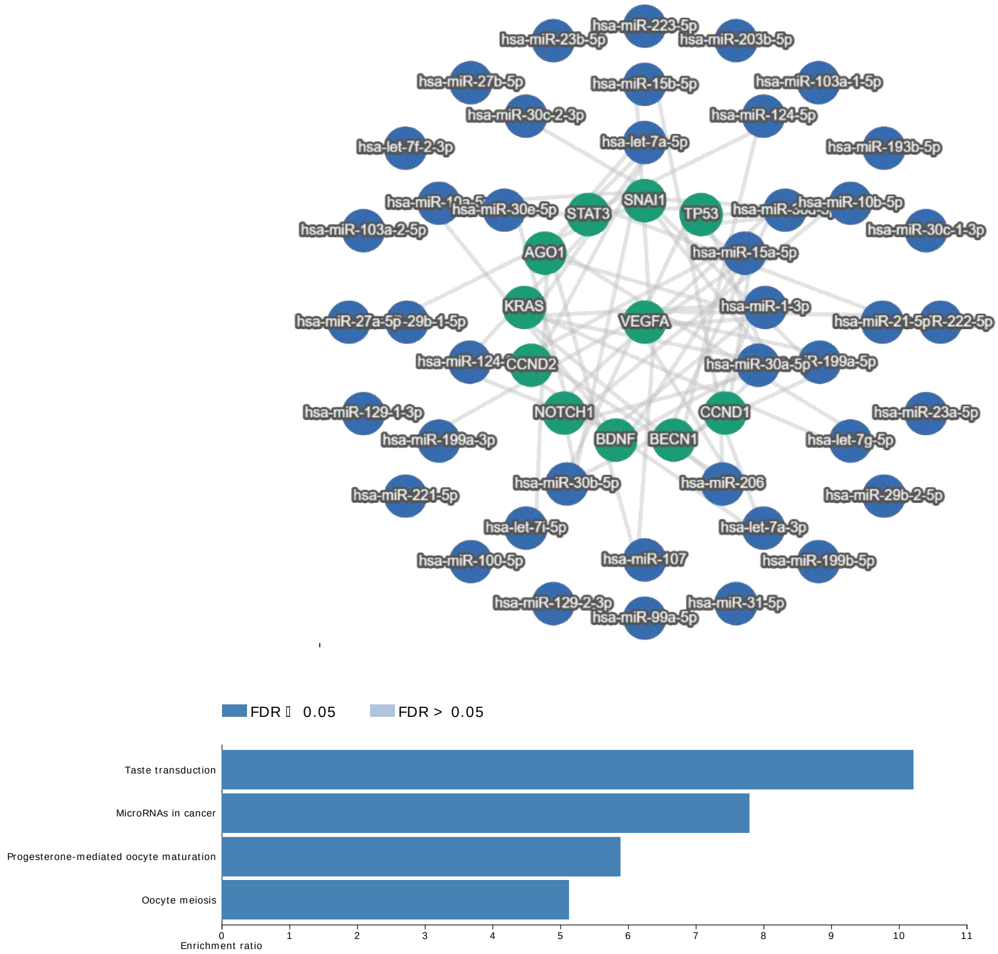

A

B

Figure S1. "MicroRNAs in cancer" interaction graph. **A,** the network has been generated through miRTargetLink 2.0 [40]. The significant microRNAs included in the KEGG "MicroRNAs in Cancer" pathway (hsa05206) are highlighted in blue, whereas their related gene targets are depicted in green. Only hits with a minimum of 5 shared validated targets genes are shown. **B,** enrichment bar plot obtained using KEGG and WebGestalt tool [41].

The heatmap plots shown below highlight the relationships between individual data points, and the corresponding relationships within clusters.

Each group has a distinct set of copy number values, and the main branches representing cancer prone and resistant organisms perfectly distinguish the species. No additional information was given to the algorithm other than copy numbers.
The model was able to discriminate between the two groups only using CNVs data. Euclidean distances, with both "complete" and "ward" methods have been applied.

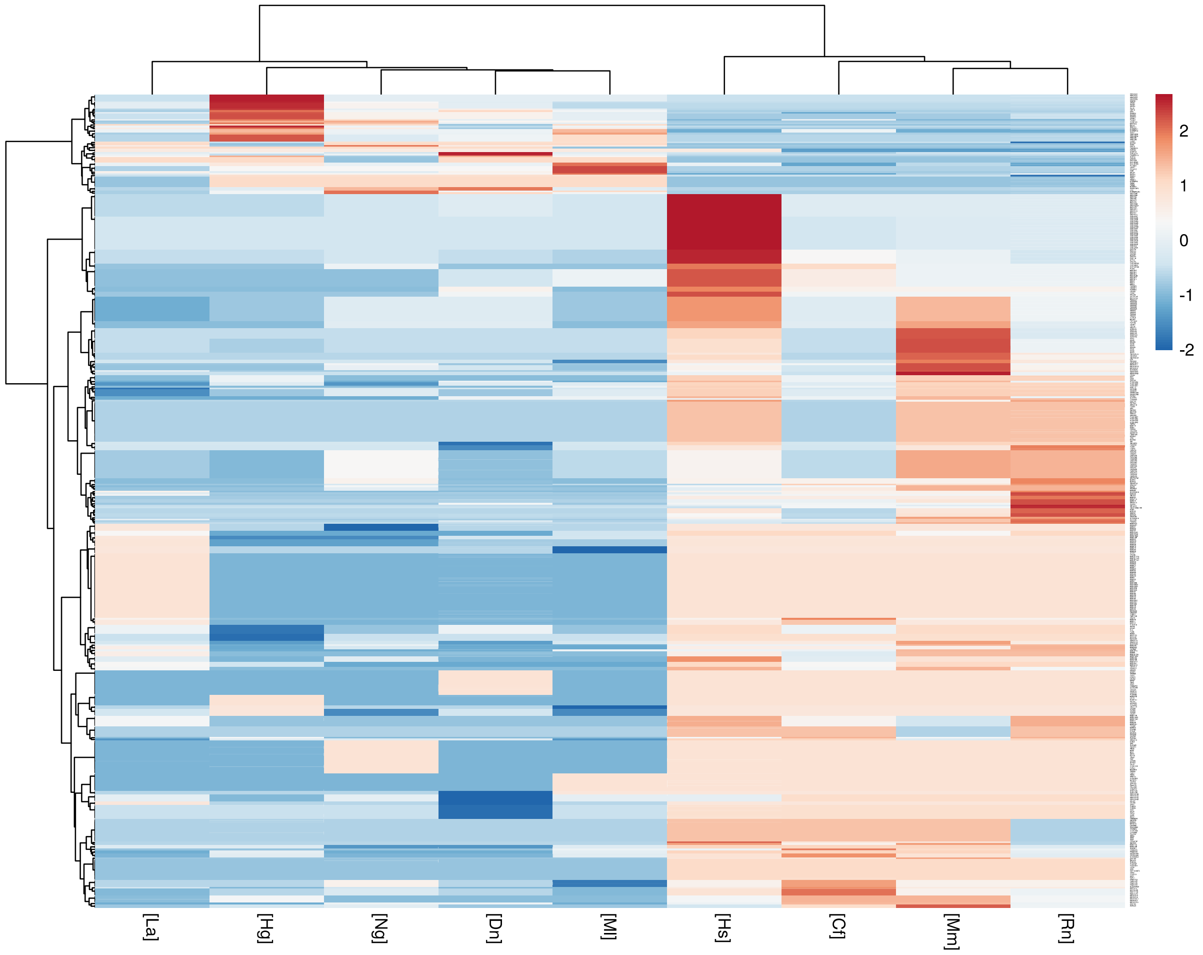

Figure S2. Heatmap of all the significant genes, clustered with Euclidean distance and ward linkage.

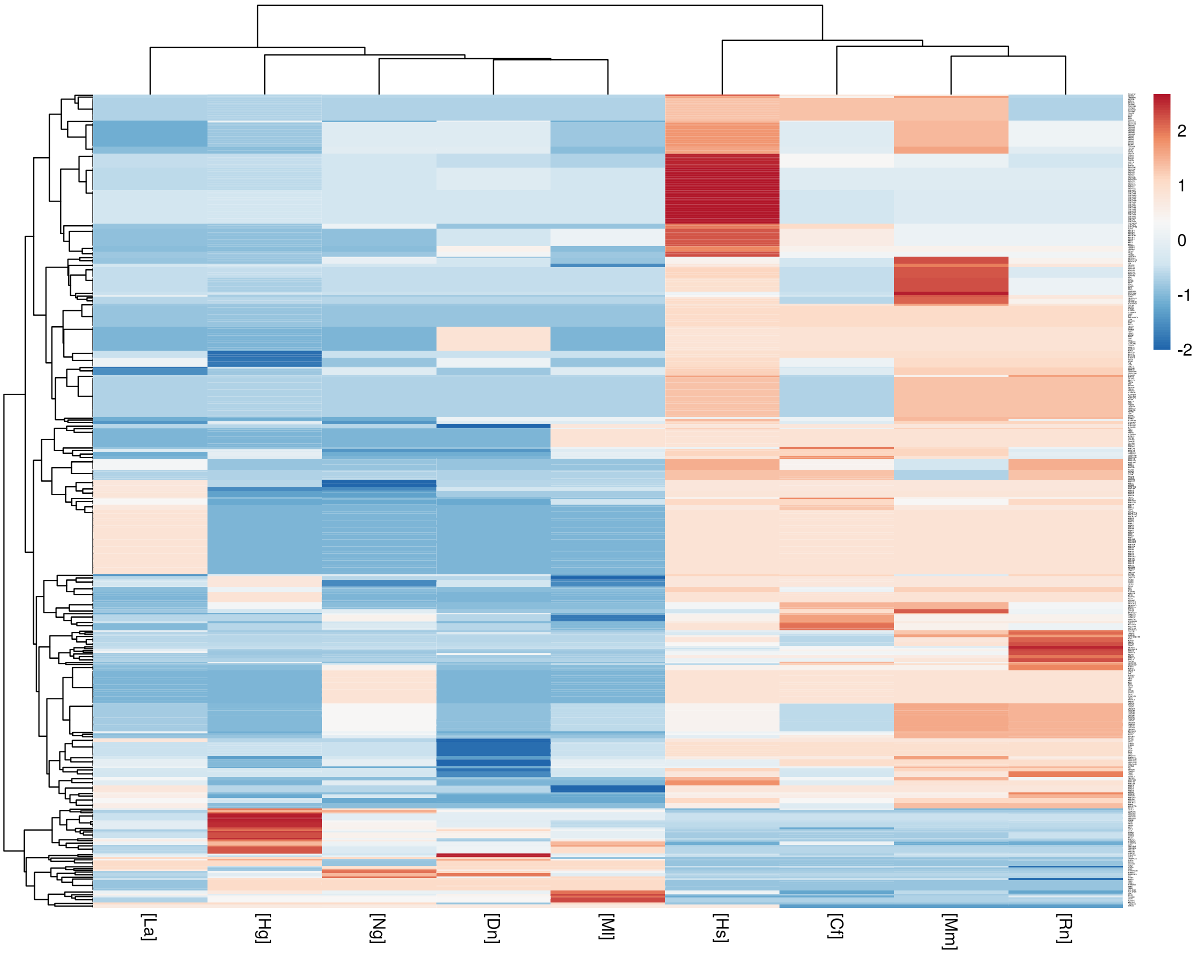

Figure S3. Heatmap of all the significant genes, clustered with Euclidean distance and complete linkage.

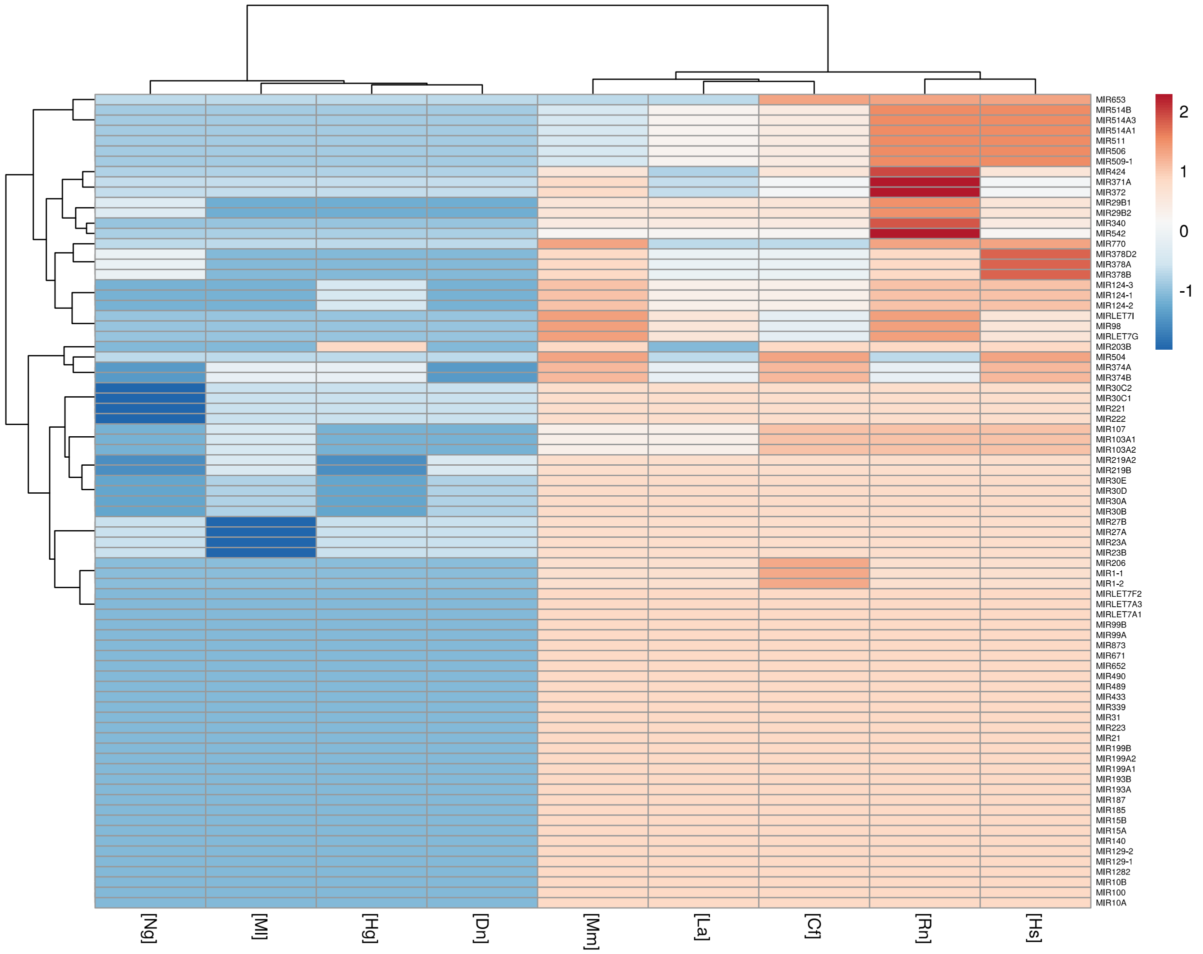

Figure S4. Heatmap of the significant MicroRNAs, clustered with Euclidean distance and ward linkage.
